# Raising Quality in Diamond Open Access

**Published:** 2026-06-15

**Authors:** Iva Grabarić Andonovski, Zrinka Pongrac Habdija, Božidar Šantek

**Affiliations:** University of Zagreb Faculty of Food Technology and Biotechnology; Food Technology and Biotechnology journal; Kačićeva 23, 10000 Zagreb, Croatia

In recent years, there has been considerable discussion about potential ways to improve the scholarly publishing system and bring research back to the community, especially when the research is publicly funded. Plan S (*1*) has made an impact on the development of national and institutional policies in the European Research Area (ERA). The goal is to develop more sustainable and equitable models in scholarly publishing by promoting the Publish-Review-Curate (PRC) model, diamond open access (OA), and the use of preprints.

Diamondisation is in the focus of the European Commission, based on the Action Plan for Diamond Open Access (*2*), supported by the CRAFT-OA (*3*) and DIAMAS (*4*) projects, which have strengthened the scholarly publishing infrastructure and provided guidelines and standards for diamond scholarly publishing. One of the key outputs of the DIAMAS project is the Diamond Open Access Standard (DOAS), a set of criteria for institutional publishers on seven core components: (*i*) funding, (*ii*) legal ownership, mission and governance, (*iii*) open science, (*iv*) editorial management, editorial quality and research integrity, (*v*) technical service efficiency, (*vi*) visibility, communication, marketing and impact, and (*vii*) equity, diversity, inclusion and belonging (EDIB), multilingualism and gender equity (*5*). Another output is the DOAS self-assessment tool, which helps publishers and editors to identify strengths and weaknesses in their diamond OA journals (*6*). The tool consists of two modules, one evaluates the alignment of a journal with DOAS, and the other evaluates the sustainability of the diamond OA publishing model.

Food Technology and Biotechnology has been using the DOAS self-assessment tool since November 2025. The first result showed 74 % alignment with DOAS, and the second evaluation in April 2026 showed an increase to 82 % (*7*). This is a result of several initiatives: implementation of the Open Science Policy of our publisher (University of Zagreb Faculty of Food Technology and Biotechnology) into journal instructions to authors (*8*), inclusion in the OpenAIRE Publisher Dashboard to improve the journal visibility (*9*), endorsement of Sex and Gender Equity in Research Guidelines (SAGER) (*10*) and introduction of EASE SAGER Checklist (*11*) into the submission process to raise the awareness about reporting on sex and gender in the research, and introduction of EASE Interactive checklist (*12*) into the submission process to help collecting the data about authors’ contributions and conflicts of interest, ethics approval for the research, and use of AI tools, among others.

Within the diamond OA publishing model, long-term sustainability depends largely on stable institutional or governmental funding rather than author-pays solutions. The journal currently relies solely on institutional funding, and we need to secure additional financial support to meet these DOAS criteria.

The journal has adopted new by-laws from July 2025, which regulate more efficiently the roles, responsibilities, mandates (3+3 years), and election procedures of editorial bodies (*13*). The new Editor-in-Chief, Professor Božidar Šantek, a member of our Editorial Board since 2009 and Deputy Editor since 2024, was appointed along with four Deputy Editors: Associate Professor Ivona Elez Garofulić, Professor Draženka Komes, Professor Blaženka Kos, and Professor Ivana Rumbak. Their appointments took into account the fields they cover and the inclusion of more women in leading roles.

Besides that, the instructions to authors and reviewers, peer reviewer form, and guidelines for the responsible use of AI tools were developed further, and Turnitin v. 2.0 AI detection tool was integrated into the journal FTB Comet online submission system, which all enable a better preparation and selection of manuscripts, and increase integrity and transparency of the published research.

As a result of all these activities, Food Technology and Biotechnology journal was successfully certified at the Hrčak Portal of Croatian Scientific and Professional journals (*14*) and received two certificates: aligned with the Hrčak criteria and aligned with the Diamond Discovery Hub criteria (*15*).

Journal efforts to raise the quality and visibility of the published content are also reflecting on the increasing number of submissions. The journal received 549 submissions in 2025, of which 28 manuscripts were accepted, 479 (87 %) were rejected, 18 were withdrawn, and 24 manuscripts are still under evaluation. In four issues, with a total of 552 pages, 46 manuscripts and two editorials were published. Issue 2/2025 was a special issue entitled Turning Agricultural Waste into Useful Biochemicals and Biofuels through Biochemical Engineering and Biotechnological Processing (Guest editor Dr Lim Jun Wei, University of Petronas, Malaysia). Submitted manuscripts were evaluated by 242 Field editors and reviewers (Acknowledgement was published in issue 4/2025).

The latest issue 1/2026 of our journal is dedicated to our late Editor-in-Chief Professor Vladimir Mrša, who edited the journal for 15 years (Guest editors: Professor Emeritus Peter Raspor (University of Ljubljana), Professor Igor Stuparević and Professor Renata Teparić (both from our institution)). The newly released SCImago Journal & Country Rank (*16*) shows that Food Technology and Biotechnology is steady in Q2 with SJR=0.551 and H-index=88. The new Impact Factor will be released by the end of June, and we will see if this trend is the same (current IF=2.5). However, we are not measuring our success with traditional metrics. Our interest is in implementing best practices in diamond OA publishing, especially open science practices, EDIB, and promoting the Sustainable Development Goals, as we are a signatory of the UN SDG Publishers Compact (*17*). We hope that we will be able to continue our diamond path and, with our new lead, improve the quality and visibility of the published content, and thus increase the trust in science.
